# Decontamination technologies for medicinal and aromatic plants: A review

**DOI:** 10.1002/fsn3.2707

**Published:** 2022-01-11

**Authors:** Edris Rahmati, Mohammad Hadi Khoshtaghaza, Ahmad Banakar, Mohammad‐Taghi Ebadi

**Affiliations:** ^1^ Department of Biosystems Engineering Tarbiat Modares University Tehran Iran; ^2^ Department of Horticultural Science Tarbiat Modares University Tehran Iran

**Keywords:** bioactive composition, food safety, herb and spices, microbial contamination, thermal and nonthermal processing

## Abstract

Microbial quality assurance has always been an important subject in the production, trade, and consumption of medicinal and aromatic plants (MAPs). Most MAPs have therapeutic and nutritional properties due to the presence of active substances such as essential oils, flavonoids, alkaloids, etc. However, MAPs can become infected with microorganisms due to poor hygienic conditions during cultivation and postharvest processes. This problem reduces the shelf life and effective ingredients of the product. To overcome these problems, several technologies such as using ethylene oxide gas, gamma irradiation, and steam heating have been used. However, these technologies have disadvantages such as the formation of toxic by‐products, low consumer acceptance, or may have a negative effect on the quality of MAPs. This requires a need for novel decontamination technology which can effectively reduce the biological contamination and minimize the food quality losses. In recent years, new technologies such as ozonation, cold plasma, ultraviolet, infrared, microwave, radiofrequency and combination of these technologies have been developed. In this review, biological contamination of MAPs and technologies used for their decontamination were studied. Also, the mechanism of inactivation of microorganisms and the efficacy of decontamination techniques on the qualitative and microbial characteristics of MAPs were investigated.

## Graphical abstract

Medicinal and aromatic plants are often contaminated with biological contamination. Usage of novel technologies is different according to the type of product. Combined systems were suggested to be a promising technique for decontamination.

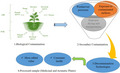

## 1 INTRODUCTION

The use of medicinal and aromatic plants (MAPs) dates back to the ancient Egyptians and today they are used in most countries in the food and pharmaceutical industries. MAPs, due to their active substances such as essential oils, flavonoids, alkaloids, minerals, and nutrients, have therapeutic and nutritional properties with regard to their structure (Barata et al., 2016; Embuscado, 2015; Yilmaz, 2020). These properties are obtained from different parts of the plant, such as leaves, roots, flowers, fruits, and seeds (Saha & Basak, 2020). According to the World Health Organization (WHO), approximately 60% of the world's population rely on these plants and the demand on their derivatives is increasing noticeably because of their nutritional and medicinal values (Kiani et al., 2016; Shrestha & Dhillion, 2003).

MAPs are considered as one of the most important strategies with respect to the health and trade in all countries of the world. So, their exports play a chief role in a country's economy. According to the International Trade Center, MAPs are traded annually in the world for 4 billion dollars and are anticipated to grow to about 6.5 billion dollars in the near future (Jack, 2006). Global imports of MAPs in 2016 were reported at 660,261 metric tons worth $ 2.9 billion and exports at 537,149 metric tons worth $ 3 billion. China, India, Canada, the United States, and Germany, with about 60% of global exports, are the main suppliers of MAPs. Germany, the United States, China, Japan, and Singapore also accounted for 50% of global imports (Nguyen et al., 2019). Currently, the most important MAPs for world trade are black pepper, cardamom, vanilla, cloves, ginger, cinnamon, cassia, and turmeric found in the tropics, and coriander, cumin, sage, thyme, and mints in the nontropical regions (Embuscado, 2015).

MAPs, like other agricultural products, may be exposed to a wide range of contaminants, such as pesticides and herbicides, heavy metals, biological contamination, polycyclic aromatic hydrocarbons, and other environmental contaminants (Chan, 2003; Smith‐Hall et al., 2012). Studies have shown that biological and chemical aspects share the greatest part of contamination. Biological contamination of MAPs such as fungi, yeast, viruses, bacteria and their spores, and insects (living or dead) can occur at any stage during production and marketing (Chmielewski & Migdał, 2005; de Freitas Araújo & Bauab, 2012). Factors that lead to an increase in these contaminants include the delay in drying time, improper drying, postharvest processes, exposure to contaminated surfaces, inappropriate transportation, and moisture absorption during storage. Storage and processing conditions basically determine the quality of the final MAPs. These problems are more prevalent in tropical and subtropical regions, because the high temperature and moisture contents are favorable to fungal growth and toxin production (Zhang et al., 2012). Microbial contamination of MAPs is an important subject with respect to consumer safety, negative impacts on active substances, and nutritional properties of these plants, their export, and the quality standards of importing countries. Additionally, it reduces the shelf life of the product and also the accumulation of mycotoxins (Kneifel et al., 2002; Kosalec et al., 2009; Stępień et al., 2011; Waśkiewicz et al., 2008).

Mycotoxins are secondary metabolites that are formed by a wide range of contaminating fungi in a variety of foods and agricultural products around the world and potentially endanger human health (Science, 2003). According to the Food and Agriculture Organization of the United Nations, about 25% of the world's crops have been contaminated by mycotoxins during growth or storage (Wu, 2007). Therefore, the microbial load in the product must be minimized or completely eliminated. Although the use of preventive measures such as good manufacturing practice (GMP) and guidelines on good agricultural and collection practices (GACP) can control these contaminants, the necessary infrastructure has not yet been provided in many areas of the world (Kosalec et al., 2009). Considering that medicinal plants and spices are collected from different areas of the world, their quality control and microbial safety play an important role in their trade and consumption. A lot of equipment and techniques have been employed to decontamination of medicinal and aromatic plants and their derived products. The most common commercially available methods for decontaminating MAPs are ethylene oxide and methyl bromide, heat treatment, and gamma irradiation.

Ethylene oxide and methyl bromide fumigation is currently banned in the United States and the European Union due to the formation of toxic and carcinogenic by‐products (Sánchez‐Maldonado et al., 2018; Shirkole et al., 2020). In the gamma–irradiation method, the maximum dose used should not exceed 10 kGy, as it endangers the health of consumers and can also cause structural damage to the food products such as, odor, color, flavor, and the reduction of volatile compounds regarding these technologies. Also, high cost of the decontamination process, formation of radioactive materials in packaged products, and a poor consumer acceptance toward irritated foods have been reported (Cho et al., 2017; Verma et al., 2021). Steam treatment also has adverse effects on physicochemical qualities, nutritional properties, and quality parameters of the product. Furthermore, it requires a heat treatment step because of dampened surface of the product, which does necessitate high energy consumption (Cheon et al., 2015; Molnár et al., 2018; Schweiggert et al., 2007). This requires a need for a new decontamination technology that can effectively reduce the biological contamination and minimize the food quality losses.

In recent years, new methods include: physical (ultraviolet, cold plasma), chemical (ozone), and thermal methods (infrared, microwave, and radiofrequency) or a combination of them (combination of two or more technologies to achieve synergistic effects) which have been used by the researcher. Novel technologies for decontamination have attracted the attention of a lot of food manufacturers. Some new technologies are all under research in laboratories, while other novel technologies are still undergoing initial testing. To the best of our knowledge, no comprehensive research has been done on the equipment and systems used to decontaminate MAPs. Hence, the path taken in this article is to review the technologies used in MAPs’ decontamination and their effects on the physicochemical and microbial properties of MAPs.

## 2 COMMON METHODS FOR DECONTAMINATION

### 2.1 Ethylene oxide and methyl bromide injection

Ethylene oxide and methyl bromide treatment is a decontamination method that has been commonly used to decrease microbial infection in MAPs due to its efficiency and relatively low cost. Despite the above advantages, this method was banned for the formation of toxic by‐products, carcinogenicity, safety, and environmental issues in 1991 by the European Union and many other countries (Schweiggert et al., 2007; Asill et al., 2013).

### 2.2 Gamma irradiation

Irradiation is the amount of energy required for ionization that is transferred from the radiation source (^60^Co or ^137^Cs) to the food. Its main mechanism of inactivation of microorganisms is damaging the DNA of the cell (Khawory et al., 2020). Gamma irradiation was approved in 1983 by the Codex Alimentarius Commission (CAC) for microbiological decontamination of MAPs and is currently used in at least 51 countries up to a maximum dosage of 10 kGy (Khawory et al., 2020). One of the main advantages of gamma irradiation is its effective penetration depth and also its power to decontaminate the internal parts of the product. Numerous studies have shown the positive effects of gamma irradiation on reducing the microbial load of MAPs (Al‐Bachir, 2007; Al‐Bachir et al., 2004; Kamat et al., 2003). Irradiation reduces the damage caused by microbial contamination and insects. In addition, this method is fast, convenient, and user‐friendly. As well, the chance of recontamination of MAPs reduces because disinfestation takes place after packaging (Farkas, 1998; Khattak & Simpson, 2009). Gamma irradiation can be used at controlled doses, and higher doses can only be used in certain cases. In other words, in gamma irradiation, the maximum amount of absorption for food should not exceed 10 kGy, as it endangers the health of consumers and affects the structural and sensory characteristics of food. However, irradiation has been reported to have disadvantages such as potential impacts on the quality of MAPs, the high cost of the process, the formation of radioactive materials in packaged products, and the general lack of acceptance of products by consumers (Akbas & Ozdemir, 2008; Ban et al., 2018; Chytiri et al., 2005; Gumus et al., 2011).

### 2.3 Steam heating

**FIGURE 1:**
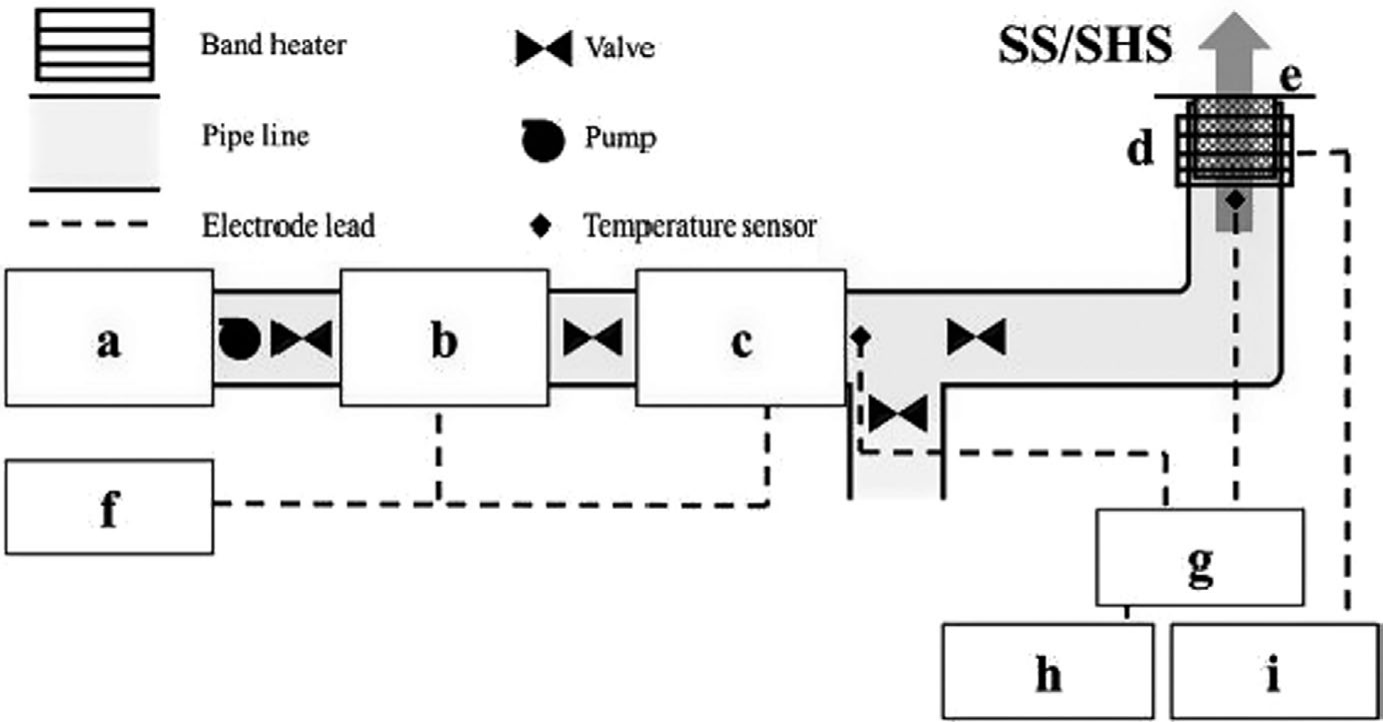
Schematic diagram of the custom‐made saturated steam (SS)/superheated steam (SHS) decontamination machinery. (a) Water reservoir, (b) steam boiler, (c) superheater, (d) outer reacting chamber unit, (e) inner reacting cell, (f) power control unit, (g) temperature monitoring system, (h) temperature data processing system, and (i) temperature controller for the band heater

The steam heating system has been successfully used in the MAPs’ industries for decontamination. Depending on the operation temperature, steam heating systems are divided into two categories: saturated steam (SS) and superheated steam (SHS). Saturated steam is a common method used for decontaminating spices in the United States and Europe in two ways: continuous or batch (Abba et al., 2009; Schweiggert et al., 2007). The general schematic of the saturated steam/superheated steam decontamination machinery with its components is shown in Figure (1).

The amount of steam produced in this system varies according to the inlet power to the system (inlet power to the boiler and superheater) and the capacity of the boiler. The working mechanism of this method is executed on the basis of steam use at a temperature of 100–200°C. For this purpose, the steam flow conveys heat to the surface of the product (convective heat transfer) to increase the temperature of the product and also to decontaminate it. Microbial contamination in plants has been reduced greatly with respect to temperature and time treatments, and gradually settled within the standard range. However, treatment with steam heating system encounters some disadvantages such as high energy consumption, complexity of equipment, color and sensory alterations, and reduction of volatile compounds (Ban & Kang, 2016; Brodowska et al., 2014; Rico et al., 2010; Tateo & Bononi, 2006; Waje et al., 2008).

There are some companies which manufacture steam systems to decontaminate MAPs, such as Napasol AG (Rotosol^®^ and Statisol^®^), Ventilex^®^ (Ventilex continuous steam sterilizing system), Log5^®^ (continuous HT‐ST “In‐Flow” steam TEMA Process BV decontamination process), ETIA (Safesteril^®^), Revtech (Revtech^®^), etc. In these technologies, saturated steam is used for decontamination, and additional equipment is taken to control condensation and the uniformity of the decontamination operation. Also, these systems are useful for decontamination of MAPs, but they are expensive. Therefore, the development of new technologies is needed considering environmental issues, optimal energy consumption, and production of high‐quality goods.

## 3 NEW TECHNOLOGIES

### 3.1 Ozone injection

**FIGURE 2:**
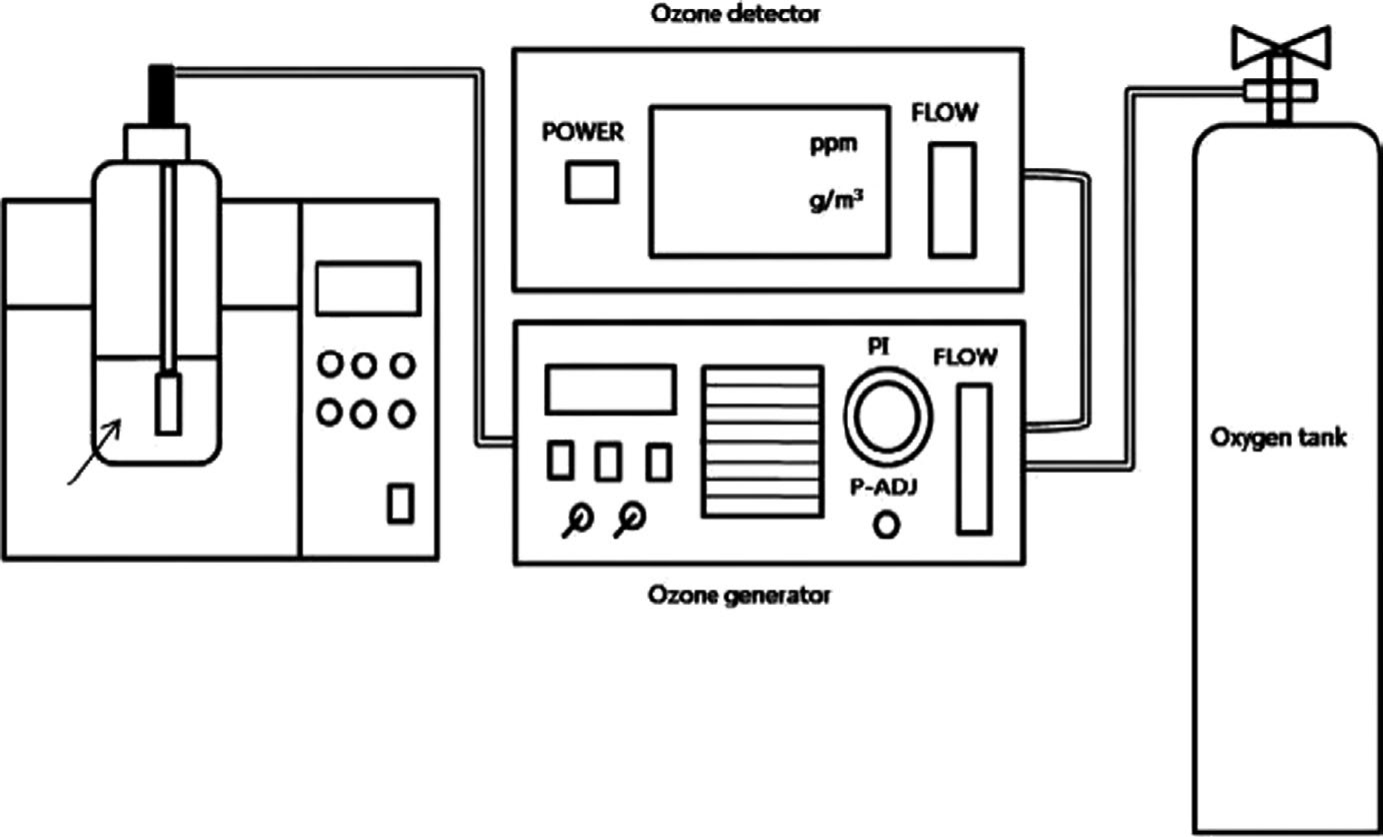
Schematic diagram of the ozone system

The use of ozone in 2001 in both gaseous and water‐soluble forms has been approved by the US Food and Drug Administration as a strong oxidation for decontamination and food processing (Khadre et al., 2001). Using ozone as a safe method, to inactivate microorganisms, has been increased with respect to the food industry, especially for fluid foods. The schematic diagram of the ozone treatment system (water‐soluble) is shown in Figure (2). The system consists of four main parts: an oxygen chamber, an ozone detector, an ozone generator, and a treatment chamber. For this purpose, all the oxygen molecules enter the generator and decompose to single oxygen molecules during reactions by ultraviolet (UV) irradiation, then the oxygen atoms react with each other to form ozone molecules (Mohammadi et al., 2017). The produced ozone is then injected into the chamber and the decontamination process triggered subsequently. Ozone antimicrobial property is associated with the oxidation of double‐bond cellular compounds such as phenolic rings and sulfhydryl groups, which ultimately lead to cell death (Aponte et al., 2018; Pandiselvam, Mayookha, et al., 2020). Ozonation has been reported to decrease the microbial load on food powders (Akbas & Ozdemir, 2008; Ha & Kang, 2014; Pandiselvam, Subhashini, et al., 2019; Proctor et al., 2004; Tiwari et al., 2010). Decontamination efficiency with ozone technology is affected by factors such as the type of microorganisms, the amount of microbial contaminations, temperature, pH medium, relative humidity, additives, and the amount of organic matter around the cell (Han et al., 2002; Kim et al., 1999; Manousaridis et al., 2005). However, the main disadvantage of this technique is the potential toxicity of ozone molecules to the operator. Therefore, the decontamination process should only be performed in an isolated and well‐ventilated chamber. Ozone should also be allowed to be decomposed to oxygen molecules, which usually takes 20 to 50 min at room temperature (Skåra & Rosnes, 2016; Thanushree et al., 2019).

### 3.2 Cold plasma

**FIGURE 3:**
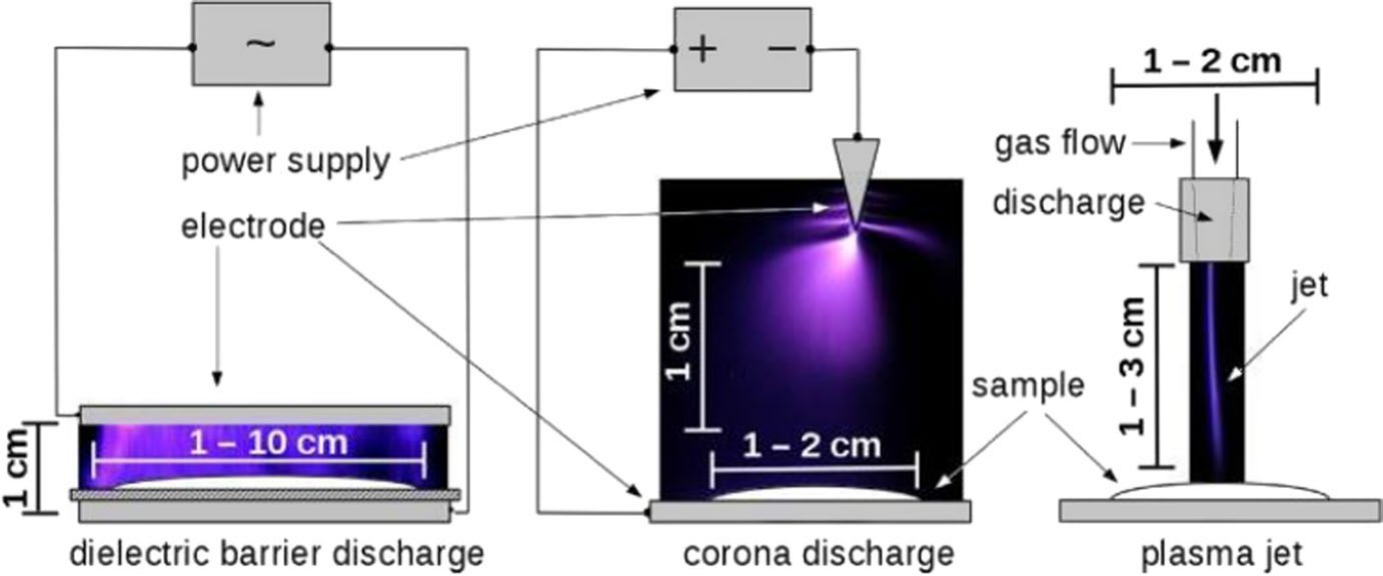
Schematic diagram of electrical discharges for generating the cold plasma

Since the mid‐1990s, plasma has been used to inactivate microorganisms. However, this method has recently been studied in the food industry (Li & Farid, 2016). In general, plasma is of two types: cold plasma and thermal plasma. Cold plasma is a relatively ionized gas that is coupled from energy sources such as corona discharge, dielectric barrier discharge (DBD), microwave discharge, pulse discharge, high‐frequency discharge with gaseous medium such as nitrogen, oxygen, air, hydrogen, halogen, argon, or combination of them (Sakudo et al., 2020; Scholtz et al., 2015). Common electrical discharge equipment for the generation of cold plasma is shown in Figure (3). Reactions of various plasma compounds such as free radicals, charged particles, ultraviolet photons, ions, and heat lead to the oxidation of microbial cell membrane, DNA alteration, and thus inactivating microorganisms (Gallagher et al., 2007; Laroussi & Leipold, 2004; Lee et al., 2015). Cold plasma is a relatively fast, environmentally safe, and low‐temperature processing method that has been successfully recruited to inactivate microorganisms of MAPs (Kalkaslief‐Souza et al., 2007; Kim et al., 2014; Pankaj & Keener, 2018). Plasma decontamination efficiency depends mainly on the type of gas, voltage, the energy source, gas composition, treatment time, type of product, and the relative humidity (Li & Farid, 2016; Patil et al., 2014).

However, cold plasma has disadvantages such as poor penetration capacity into food (especially solid foods) and unavailability on a commercial scale. In addition, information about its impact on the quantity and quality of active substances of product is limited (Ebadi et al., 2019).

### 3.3 Ultraviolet irradiation

**FIGURE 4:**
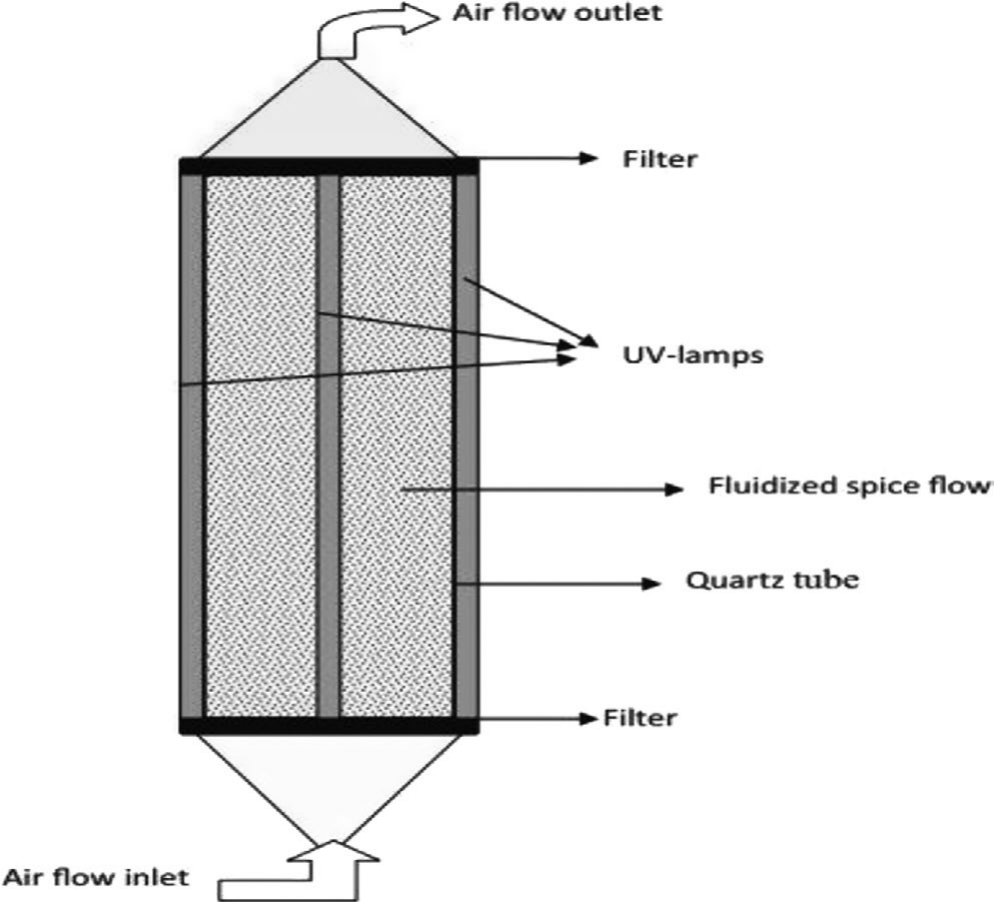
Schematic diagram of the ultraviolet (UV‐C) system

Ultraviolet irradiation was first used in France in 1906 to decontaminate beverages (Li & Farid, 2016). Ultraviolet is a nonthermal technology that uses electromagnetic spectrum (100–400 nm) to inactivate microorganisms. Its antimicrobial effect has been effectively confirmed within the range of 200–280 nm (ultraviolet‐C) (Pedrós‐Garrido et al., 2018). Inactivation of microorganisms comes from the ability of ultraviolet to penetrate the cell membrane and to damage the DNA or RNA of microorganisms, thus preventing their proliferation (Escalona et al., 2010; Gabriel, David, et al., 2020). This method can potently inactivate microbes and has recently been successfully used to decrease the microbial load of solid food samples (Fonseca & Rushing, 2006; Gabriel et al., 2017; Pérez‐Gregorio et al., 2011). Decontamination efficiency by ultraviolet depends on factors such as the type of microorganisms, ultraviolet dose, and temperature of the treatment and the surface characteristics of the food (Fan et al., 2017). Failure to expose the total and effective surface of the product to ultraviolet and nonuniform decontamination operations are among the disadvantages of ultraviolet irradiation. To solve this problem, it is proposed to use a combined fluidized bed system with ultraviolet (Figure 4). However, this method has a low penetration depth and its application is limited to inactivating surface microorganisms (Guerrero‐Beltr·and Barbosa‐C·novas, 2004).

### 3.4 Infrared radiation

**FIGURE 5:**
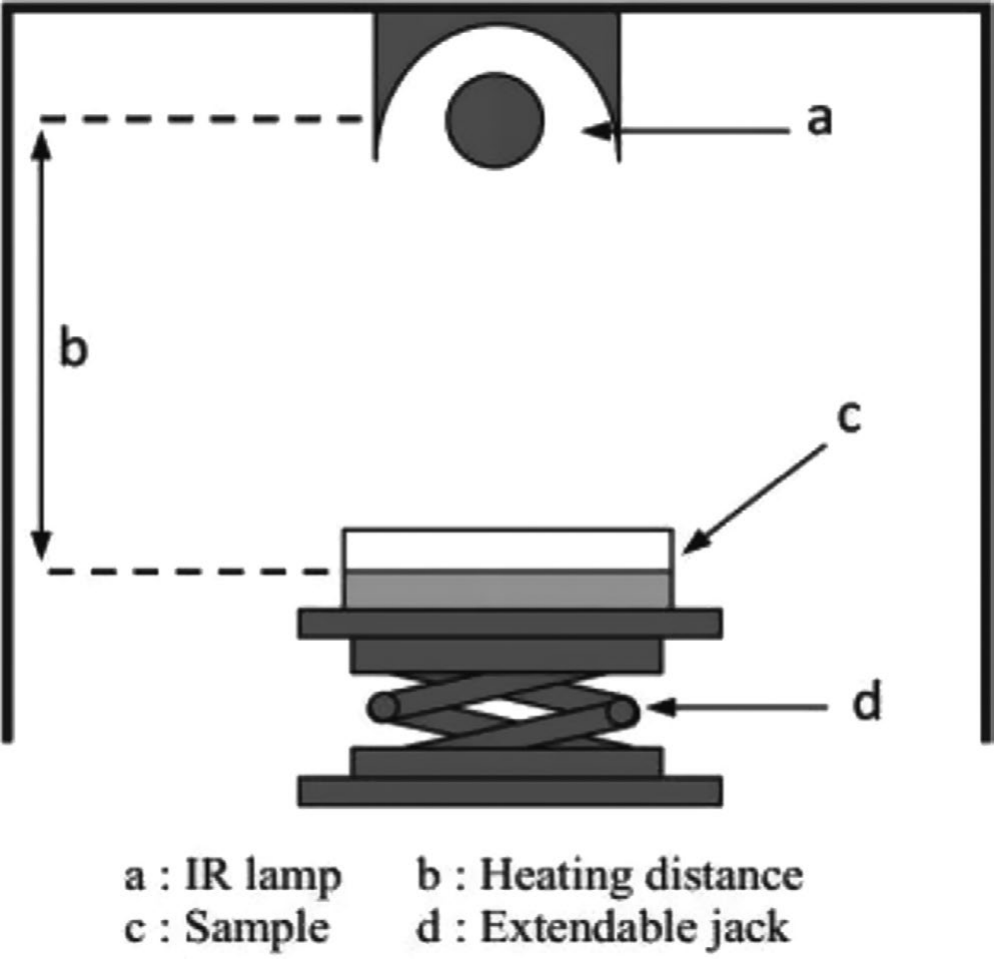
Schematic of infrared (IR) heating equipment

In recent years, infrared radiation (IR) has been studied as a new technology for MAPs’ decontamination. IR is a region of electromagnetic spectrum with a wavelength in the range of 0.76 μm–1 mm between ultraviolet and microwaves. IR is generally divided into 3 spectral regions: near‐infrared sections (0.76–2 μm), mid‐infrared (2–4 μm), and far‐infrared (4–1000 μm) (Eliasson et al., 2015). In general, far‐infrared is used for food processing because most food compounds absorb radiant energy within this range. Infrared radiation is absorbed by organic compounds in low‐water activity foods such as protein, lipids, and sugars. The mechanism of infrared inactivation takes place through the absorption of energy by organic food compounds and damage to DNA, RNA, and proteins in microbial cells (Gurtler et al., 2014; Rifna et al., 2019; Sandu, 1986). The schematic of the IR heating system is shown in Figure (5). Several studies had examined the antimicrobial effects of infrared radiation (Bingol et al., 2011; Brandl et al., 2008; Eliasson et al., 2015; Erdoğdu & Ekiz, 2013; Ha et al., 2012). Infrared decontamination efficiency depends on parameters such as decontamination temperature, infrared power, distance from the source, etc. (Rifna et al., 2019). Infrared heating has a penetration depth of 0.31–4.76 mm depending on the product and the wavelength used, so infrared heating is considered as a surface heating technology (Eliasson et al., 2015). However, infrared radiation has not been widely used as an alone energy source.

### 3.5 Microwave heating

**FIGURE 6:**
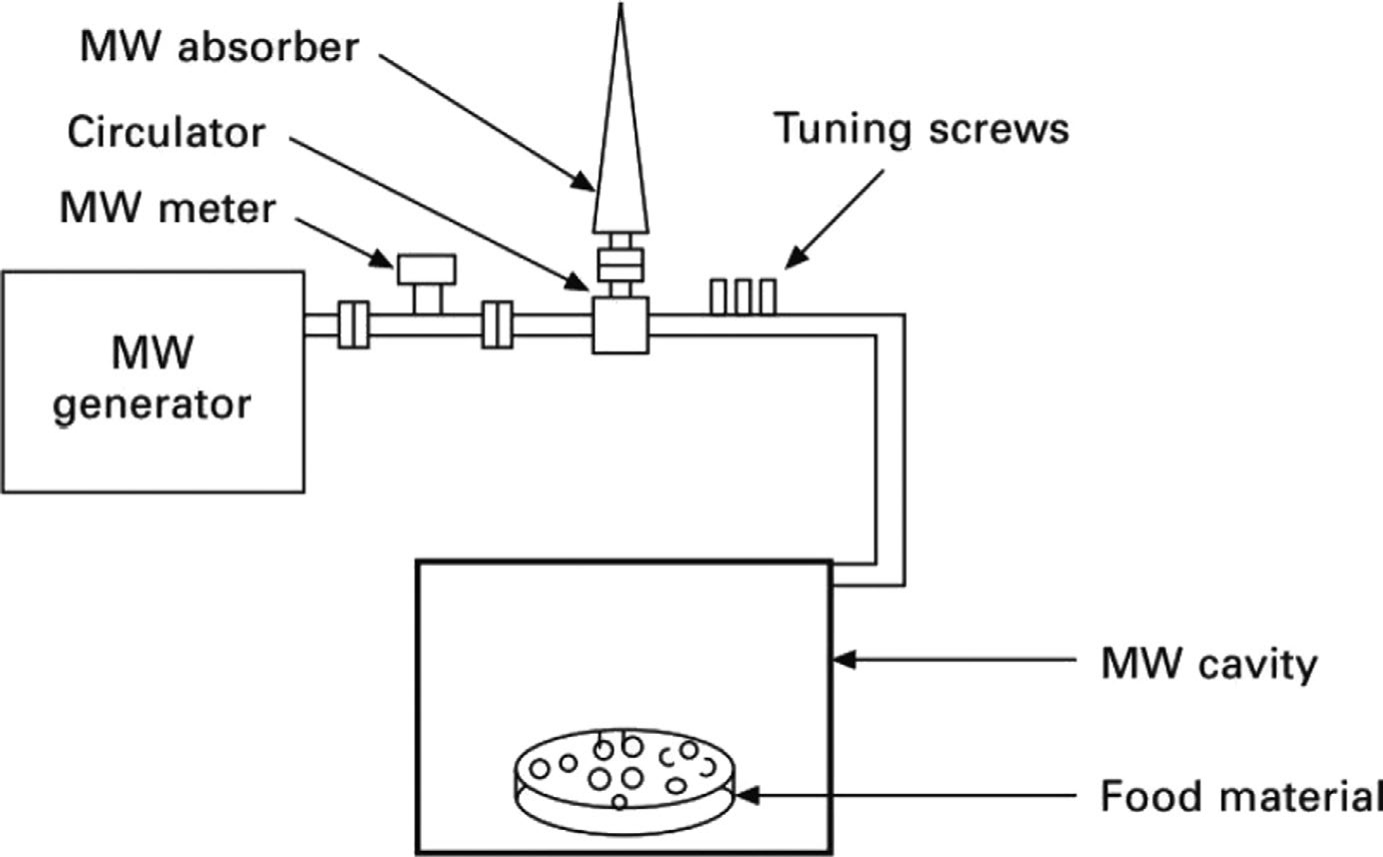
Components of an industrial microwave heating system

Microwaves are a type of nonionizing radiation of electromagnetic waves with wavelengths and frequencies in the range of 1 mm–1 m and 300 MHz–30 GHz, respectively. In general, two frequencies, 915 and 2450 MHz, have been employed for medical, industrial, and scientific applications (Pankaj et al., 2018). The wavelength related to these frequencies is in the range of 12–24 cm. A schematic of a microwave heating system is shown in Figure (6). A conventional microwave heating system consists of three main components: a magnetron, a waveguide, and a sample chamber (Ştefănoiu et al., 2016). Electromagnetic waves are generated by a magnetron and transmitted to the surface of the product via a waveguide.

The interaction of microwaves with the sample depends on the wave characteristics (frequency and wavelength) and the nature of the sample. Microwaves penetrate foods and generate heat inside the product. Due to the interaction of electromagnetic waves with the sample, the heat is generated in the food via dipolar rotation and ionic conduction. In microwave heating, the major mechanism of heat generation is the rotation of dipoles, in which the dipole molecules rotate in the presence of an electromagnetic field and the friction caused by them leads to the generation of heat. The amount of heat generated is a function of the electromagnetic frequency, dielectric properties of the material, and the intensity of the electric field (Dev et al., 2012). In microwave heating, the mechanism of inactivation of microorganisms is based on the electroporation of cell membranes, which, based on the intensity of the electric field used, leads to the formation of reversible or irreversible pores (Palgan et al., 2012).

For the volumetric heating mechanism and low processing time, microwave heating (dielectric heating) has been extensively hired in pasteurization and sterilization of food. The microwave sterilization process takes place in the temperature range of 110–130°C. This method effectuates more preservation of nutrients, vitamins, and aromatic compounds in solid foods compared to conventional heat treatments. The following is a summary of research on decontamination using a microwave heating system.

Eliasson et al. (2015) examined two methods of microwave heating and infrared heating (98°C for 20 min) in order to reduce the microbial load of paprika powder. The results showed that microwave and infrared treatments reduced the total number of mesophilic bacteria by 4.8 and 3.8 log units, respectively.

Jeevitha et al. (2016) investigated the effect of different microwave treatments such as microwave power (663 and 800 W), treatment time of 1–15 min, and moisture content (110 and 260 g/kg on a wet basis) on the microbial load of black pepper. The results showed that the power level of 663 W for 12.5 min reduces the microbial load down to the standard level. Eventually, it was reported that microwave heating can be recruited effectively to lessen the microbial load of black pepper without affecting its quality properties.

Behera et al. (2017) investigated the effect of different microwave treatments such as microwave power density (10, 33.5, and 57 W/g), treatment time (10, 20, and 30 s), and product thickness (1, 2, and 3 mm) on the microbial and physical properties of dried turmeric powder. The results showed that the decrease of more than one log in the total number of yeast and molds leads to a significant reduction in the product's moisture. Microwave power density significantly affected water activity, final product temperature, and the number of yeast and molds. However, the sample thickness and treatment time only exerted influence on the final product temperature, water activity, and total moisture losses. The results of numerical optimization revealed that the power density of 57 (W/g), thickness of 1.64 mm, and treatment time of 30 s bring on the optimal values of the dependent variables.

The efficacy of microwave treatment depends on factors such as treatment time, product geometry, type of microorganism, power, and frequency range hired (Jiang et al., 2018). However, microwave heating has disadvantages including nonuniform heating and the formation of cold spots inside food, which has limited the industrial use of this method for disinfection.

### 3.6 Radiofrequency heating

Radiofrequency is a type of dielectric heating, which has the potential for uniform and rapid heating of solid and semisolid samples. Radiofrequency is part of electromagnetic waves with a frequency in the range of 30 kHz–300 MHz. Usually, three frequencies of 13.56, 27.27, and 40.68 MHz are used in food processing (Ştefănoiu et al., 2016). In this method, unlike microwave heating, ionic conduction is the main mechanism of heat produced inside the product (Dev et al., 2012). The displacement of ions with opposite charges in the presence of an alternating electric field leads to an increase in the kinetic energy of the molecules and thus to an increase in the temperature of the product. In radiofrequency heating, the heat generated inside the food as a result of radiofrequency radiation is absorbed by the DNA of microorganisms, and subsequently it leads to a change in their physical structure and reduced function (Rifna et al., 2019).

**FIGURE 7:**
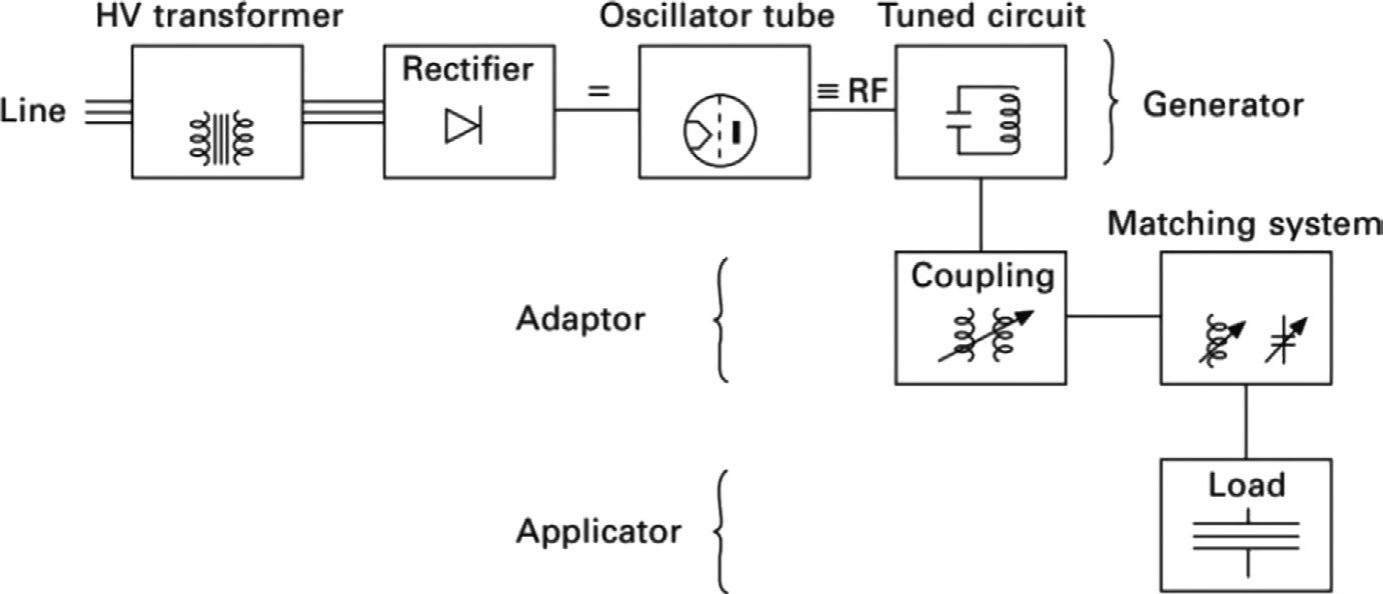
Components of a radiofrequency heating system

A schematic of the radiofrequency heating system is shown in Figure (7). A conventional radiofrequency system consists of a radiofrequency generator, an adapter, and an applicator (Dev et al., 2012). The applicator consists of two metal plates between which the product is placed. These plates (electrodes) form a capacitor with the food. In radiofrequency heating, the electrodes do not come into contact with food, so they can easily be used for solid and liquid foods. The structure of the radiofrequency heating system is simpler compared to the microwave, in addition to being able to penetrate deeper into the food because of its longer wavelengths and more uniform field patterns. Radiofrequency heating is now generally used on an industrial scale for drying processes in the textile, paper, and biscuit industries (Orsat & Raghavan, 2014). Recently, this method has been employed to decontaminate solid foods.

The following is a summary of the research carried out in the field of decontamination hiring a radiofrequency heating system.

Verma et al. (2021) investigated the effect of radiofrequency heating treatment on the microbial load and quality parameters of dried basil leaves such as color, total phenol, and antioxidant activity at three‐time levels (45, 55, and 65 s). The temperature of the samples reached 65, 80, and 100°C during the treatment, respectively. The results showed that during 55 s, the population of *Salmonella* and *E. faecium* population decreased by 4.8 and 2.7 CFU/g, respectively. Treatment for 65 s also reduced both types of microorganisms to the desired level without significant changes in product quality parameters.

Liu et al. (2021) used radiofrequency heating followed by hot air flow to control the natural microflora of Sichuan pepper. For this, radiofrequency treatment was applied at 50°C for 10 min, followed by hot air at 50°C for 10 h. In order to evaluate the desired treatments, microbial load and quality characteristics were compared with those of the control treatment (50°C for 10 h). The amount of microbial load in radiofrequency treatment and control sample was 2.45 ± 0.19 log CFU/g (d.b) and 2.95 ± 0.20 log CFU/g (d.b), respectively. However, radiofrequency treatment led to a significant reduction in color and flavor of the product. Finally, it was reported that the combination of these two methods can effectively control the microorganisms of the product, but a remarkable effect will be observed in the quality parameters of the product.

Decontamination efficiency using radiofrequency treatment depends on factors such as radiofrequency temperature, sample geometry, sample moisture, equipment capacity, and the desired microorganisms (Rifna et al., 2019). However, the dielectric properties of food change continuously with temperature, and this should be considered in the design of dielectric heating systems as it might affect the processing time and uniformity of heating. Therefore, applying this technology to food decontamination on an industrial scale requires computer simulation of temperature dependence on the dielectric properties of materials.

## 4 EFFECT OF DECONTAMINATION TECHNIQUES ON THE QUALITATIVE AND MICROBIAL CHARACTERISTICS OF MAPS

MAPs which are produced in different regions, besides their physical and chemical properties, should be evaluated for their microbial contamination. Table (1) provides an overview of the most prominent studies on the decontamination of MAPs and the impact of these methods on the qualitative and microbial characteristics of processed plants. In addition to reducing the microbial load, the qualitative characteristics of the product are affected, so selecting the most appropriate technology is of great importance for the type of product. However, an optimal relationship between the qualitative and microbial characteristics of the product is required. Therefore, along with the reduction of the microbial load, the minimization of the damage to the qualitative characteristics of the product is achieved subsequently.

**TABLE 1:** The impact of decontamination techniques on the qualitative and microbial characteristics of MAPs

Treatment condition	Processed sample	Targets	Results	Reference
*Gamma irradiation:* **‐** at 5 and 10 kGy	Dried lotus pollen	Microbial load and antioxidant properties	At 5 kGy microbial load significantly decreased also total phenol content and DPPH increased.	(Sajjabut et al., 2019)
‐ at 3–5 kGy, 5–10 kGy, 6–12 kGy and 9–13 kGy	*Gnetum gnemon, Khaya senegalensis and Euodia malayana* in two different forms (leaf extracts and dried leaves)	Bacteria, fungus, spores, total phenolic content and antioxidant activity	The results showed that the appropriate doses for extract and dried leaves were 6–12 and 9–13 kGy, respectively. Enhanced total phenolic content and antioxidant activity.	(Khawory et al., 2020)
‐ at 0, 5, 10, and 15 kGy with different atmospheres of packaging (air, *N*₂, and vacuum)	Turmeric powder	Physicochemical properties and microbial load	Decrease of 4, 4, 3, and 3 log CFU/g for coliforms, yeast/molds, total aerobic bacteria, and spore forming bacteria, respectively. Irradiation to 10 kGy at air atmosphere led to microbial safety and improved the extraction yield of bioactive compounds of the samples. Discoloration was observed in the samples irradiated (10 kGy) at different atmospheres of packaging.	(Esmaeili et al., 2018)
‐ at 0, 5, 10, 15 and 20 kGy (719 Gy/h) and following kept at temperature of (18–25°C) for 0, 6 and 12 months	Aniseed	Microbial load and sensory characteristics	Microbial load decreased to <10 aerobic bacteria per gram (>10 kGy), no significant effect was observed in color, taste, and flavor of the samples.	(Al‐Bachir, 2007)
‐at 0, 1 and 10 kGy	*Ginkgo biloba L*.	Phenolic compounds	Increase in the extractability phenolic compounds was observed in highest dose (10 kGy).	(Pereira, Barros, et al., 2015)
*Gamma rays and Electron beam:* ‐at 0, 1 and 10 kGy	*Menthapiperita, Aloysia citrodora, Melissa officinalis, and Melittis melissophyllum*	Chemical and bioactive properties	The effect of gamma rays and electron beams varied according to the type of plant. Electron beam is more effective than gamma rays.	(Pereira, Antonio, et al., 2015)
*Steam:* ‐ at 70 and 75°C for 5 min at various a_w_ (0.35–0.69)	Black peppercorns	Bacterial pathogens and color parameter	Inactivation ≥5‐log reductions achieved at all samples; no color changes were observed.	(Zhou et al., 2019)
‐ at 88 ± 5°C for 15 s–4 min	Black pepper and cumin seeds	*Enterococcus faecium and Salmonella*	>5‐log reduction of *Enterococcus faecium* and *Salmonella* in 2 min	(Newkirk et al., 2018)
‐ at 100 and 120°C, for 20 s (flash vacuum of 20 s)	Marjoram, oregano, fennel and eucalyptus	Essential oil content and microbial count	Reduced the total plate count below the detection limit (1.102 CFU/g) in fennel and eucalyptus, and also essential oil of marjoram and oregano by 93%, 59%, respectively.	(Lange et al., 2012)
‐ at 100, 120, 140, 160, or 180°C for 1–45 s	Black peppercorns	*Salmonella* and quality parameter	Temperature of 180°C for 3s was completely inactivated *Salmonella* (reduced to 6.34 CFU/g) without a deterioration in quality (moisture content, color, and texture) of peppercorns.	(Ban et al., 2018)
*Ozone:* ‐ times of 10 and 30 min in dose of 0.3, 0.6 and 0.9 ml/L	Peppermint, summer savory, Indian valerian, lemon balm and Iranian thyme	Microbial Load and essential oil	Concentration of 0.9 ppm for 30 min was the most effective in reducing the microbial load (reduction 1.12, 1.79, 3.5, and 4 log CFU/g in peppermint, summer savory, lemon balm and Iranian thyme, respectively, no effect on essential oil content.	(Asill et al., 2013)
‐ at 0.1, 0.5, 1, 5, 7 and 9 ppm for 360 min	Red pepper	Microbial inactivation and sensory characteristics	Inactivation level of 2.0 and 1.5 log numbers of *E. coli* counts and *B. cereus* (1 ppm), respectively and 1.5 log numbers of *Bacillus cereus* spores (7 and 9 ppm) were achieved, and slight changes in sensory characteristics were observed (5.0 and 9.0 ppm).	(Akbas & Ozdemir, 2008)
‐ dose of 2.8 and 5.3 mg/L with treatment time of 30, 60, 90 and 120 min	Dried oregano	Sensory properties and microbial population	Reductions of 1.8 and 2.7 log yeast and mold and aerobic plate counts, respectively (2.8 mg/L for 20 min) and 3.2 log in the aerobic plate counts (5.3 mg/L for 90 min); significant change (*p* < .05) in the appearance (5.3 mg/L for 120 min) was observed.	(Torlak et al., 2013)
‐ at 4 ppm for 30 and 60 min	Thyme, oregano, lemon verbena, mountain tea, and chamomile	Bacteria, yeast and mold	4 log reduction of oregano and 1–2 log reduction for lemon verbena, mountain tea, thyme, and chamomile (30 or 60 min of 4 ppm) was achieved.	(Kazi et al., 2018)
‐ at 2 g per hour for 10, 20, 40 and 60 min	Sumac, cumin and pepper	Microbial load and quality features	Reduced the total number of microorganisms, mold and yeast and coliform by 2, 4, and 1 logarithmic unit, respectively; significant reduction (*p* < .05) of moisture and phenolic compounds in cumin and pepper (60 min) was achieved. Reduction of DPPH in all samples was attained.	(Hemmati Moghadam et al., 2017)
*Cold plasma:* Exposed to cold plasma for 5, 15, 30, 60 and 90 min	Oregano, pepper seeds, and paprika powder	Inactivation of microbial flora and product color	>3 log10 (after 60 min) reduced microbial flora of paprika and pepper seeds; significant color changes were observed in paprika powder.	(Hertwig et al., 2015)
‐ at 300–900 W for 0–20 min, Combined treatments (70, 80, 90°C for 10, 30, and 60 min, respectively and 900 W–20 min).	Red pepper powder	*Bacillus cereus spores* and Aspergillus *flavus*	Reduction of 2.5 ± 0.3 log *A. flavus* (900 W, 667 Pa, 20 min). Combined treatments reduced 3.4 ± 0.7 log spores/g *B. cereus spores* (90°C for 30 min−900 W−20 min); combination treatment reduced color parameter significantly (*p* < .05).	(Kim et al., 2014)
‐ at 150 W for 10, 20, and 30 min	Artichoke, chamomile, ginkgo, guarana	Microbial load	Reduction of 4 and 3 logarithmic cycles for artichoke and ginkgo, respectively.	(Kalkaslief‐Souza et al., 2007)
‐ at 20, 50 and 60 W for 20 min	Dried Peppermint	Microbial load and antioxidant properties	Significant removal of *E. coli* O157:H7 at 50 and 60 W; significant increase in the total phenolic content (*p* < .05) attained.	(Kashfi et al., 2020)
‐Exposure time for 5 min	Thyme and paprika	Total count, mold and yeast	1.18 log cycle reduction of total bacterial count was attained (thyme), considerable effects were not observed for paprika.	(Rezaee, 2019)
*Microwave‐combined cold plasma:* ‐ at 20 min for 900 W and different power densities (0.17 W m^−2^ and 0.25 W m^−2^)	Red pepper	*Bacillus cereus, Aspergillus flavus*, ascorbic acid and capsaicin	*B. cereus* and *A*. *flavus spores* were reduced by 1.4 ± 0.3 and 1.5 ± 0.2 log and spores/cm^2^; concentration of capsaicin and ascorbic acid decreased.	(Kim et al., 2019)
*Cold plasma:* ‐ at 400 W for 60, 120, 180, 240 and 300 s	Whole black peppercorns	*E. coli, Bacillus subtilis, B*. *subtilis,* and *Salmonella Enteritidis*	Reduced the *E. coli, Salmonella Enteritidis, Bacillus subtilis,* and *B. subtilis* endospores to 1.0, 5.06, and 2.03 log (CFU/g) respectively.	(Mošovská et al., 2018)
*Pulsed ultraviolet:* ‐ at 0.28 J/cm^2^ pulse with flat surface and wave‐shaped surface sample holders	Black peppercorns	*Salmonella*	*Salmonella* reduced by 1.9 and 1.5 log CFU/g for wave‐shaped and flat surface holder respectively.	(Xie & Hung, 2020)
*Ultraviolet‐C:* – at 3942 mJ/cm^2^ and 13,662 mJ/cm^2^ dose	Dried bay leaves	Microbial load, color and sensory properties	Reduction of 2.70 to 3.93 log CFU/g (3942 mJ/cm^2^). No significant change was observed in color and visual sensory properties (13,662 mJ/cm^2^).	(Gabriel, Melo, et al., 2020)
*Fluidized bed ultraviolet (UV‐C):* ‐ at 16, 32, 64, and 128 min at doses 25.7, 51.4, 102.8, 205.6 J/cm^2^	Thyme	*Bacillus cereus,* mesophilic bacteria, yeast/mold, phenolic content, antioxidant capacity, and color parameter	Reduction of 0.3, 1.8 and 1.3 log CFU/g in *Bacillus cereus*, mesophilic bacteria and yeast/mold, respectively. Minor changes in color parameters were observed.	(Dogu‐Baykut and &es, 2019)
*Ultraviolet‐C:* ‐ at 2322 and 9180 mJ/cm^2^	Whole black peppercorns	Foodborne bacteria and color	Total log reductions ranged from 1.92 (*Staphylococcus aureus*) to 3.60 log CFU/g (*E. coli* O157:H7), small change observed in color.	(Gabriel, David, et al., 2020)
‐ UV 16 min at dose 4.8–10.8 J/cm^2^ and 64 min at dose 19.2–43.4 J/cm^2^	Thyme	Total aerobic mesophilic bacteria, antioxidant activity and color	Reduction of 1.38 and 1.04 CFU/g for total aerobic mesophilic bacteria in 64 and 16 min, respectively; a* values were impacted by UV‐C irradiation significantly (*p* > .05); oxidation rates were not affected.	(Dogu‐Baykut et al., 2014)
*Infrared:* ‐ at 90–100°C for 2–10 min	Oregano	*Bacillus cereus*, volatile compound, and color	Reduction of 5.6 log unit B. cereus spore (90°C for 10 min), decrease in the composition of volatile compounds, also slight change of color was observed.	(Eliasson et al., 2014)
‐ Exposed to 100, 200, and 300 W for 0−11 min	Cardamom seeds	*Bacillus cereus* and color	Reduction of 5.11 log CFU/g *B. cereus* count was obtained (300 W for 8 min); a* values were slightly affected.	(Shavandi et al., 2019)

Abbreviation: DPPH: 1, 1‐diphenyl‐2‐picr.

## 5 COMPARISON OF TECHNOLOGIES

In 2012, Germany, Turkey, Sweden, and Spain launched a joint project called GreenFoodec to overcome the limitation of conventional technologies for MAPs’ decontamination. In this project, whole black pepper (seed), paprika (powder), and oregano (herb) were decontaminated using four technologies: High pressure CO_2_ + ultrasound, cold plasma, microwave, and infrared radiation. Then, the most appropriate technology for each product was identified on the basis of physicochemical and microbial parameters of the samples. For this purpose, cold plasma was suggested for the treatment of black pepper (60 min of indirect plasma treatment), CO_2_ + ultrasound for the treatment of paprika powder (30 min high‐pressure carbon dioxide treatment at 80°C and 150 bar with a power of ultrasounds of 75V), and infrared treatment for oregano (90°C for a 10‐min holding time). Finally, it was reported that these treatment conditions could be held as a reference for other specific products. Molnár et al. (2018) examined the effect of irradiation technologies (1, 5, and 10 kGy), steam (108–125°C, 20–120 s), microwave (95°C, total treatment time 100 s), radiofrequency (95, 105, and 115°C for 50–90 s), and enhanced microwave (moisture (20, 30%), temperature (80, 95°C), time (0,10 min)) on microbial load, color, aroma, and bioactive components (carotenoids, tocopherols, vitamin C) of paprika. Irradiation has been reported to be the most effective way to reduce microbial load (2–4 orders of magnitude) followed by steam (two orders of magnitude), enhanced microwave treatment, and radiofrequency (one magnitude). However, a significant reduction in bioactive components (carotenoids, tocopherols) was observed in gamma irradiation, volatile aroma compounds in steam, and physicochemical changes in enhanced microwave treatment and radiofrequency.

Rico et al. (2010) investigated the impact of gamma–irradiation technology (10 kGy with a dose rate of 2.5 kGy/h) and steam (100°C for 16 min) on the microbial and physicochemical properties of dried red pepper during storage at ambient temperature (20 ± 2°C) and refrigerator (4 ± 2°C) conditions for 6 months. The results showed that gamma irradiation at 10 kGy dose was more effective in the reduction of initial microbial load than steam treatment with 5‐log and 1–2 log, respectively. However, gamma irradiation reduced the content of capsanthin. In addition, color changes were reported along with a decrease in sensory parameter in steam‐treated samples (room storage). Finally, gamma irradiation and refrigerated storage were proposed to minimize physicochemical changes for red pepper powder. Valiasill et al. (2017) studied the effect of two technologies of decontamination gamma irradiation (3, 7, 10, and 15 kGy) and ozone gas (0.3, 0.6, and 0.9 mg/L for 10 and 30 min) on the microbial load of *Melissa officinalis*. The results showed that ozonation had a greater effect in reducing the microbial load of *M. officinalis*. The highest effects of gamma irradiation and ozone gas were obtained at 15 kGy and 0.9 mg/L for 30 min, respectively. In the end, the use of ozone gas was reported on the decontamination of *M. officinalis* plant due to the low cost of the process and suitable disinfection techniques than gamma irradiation.

Combined systems are among the technologies that have recently been used in the decontamination of MAPs. Erdoğdu and Ekiz (2013) used ultraviolet, far‐infrared, and the combination of far‐infrared and ultraviolet technologies in the decontamination of black pepper seeds. In this research, black pepper samples were exposed to infrared radiation (300°C for 2.78 to 5.88 min, and 350°C for 1.88 to 4.33 min), followed by 2 h of ultraviolet irradiation in intensity of 10.5 mW/cm^2^. Infrared radiation for 3.5 and 4.7 min at 350 and 300°C reduced total mesophilic aerobic bacteria to the 10^4^ CFU/g, while no significant change was observed in color and volatile oils. However, no significant reduction in total mesophilic aerobic bacteria was observed by ultraviolet irradiation alone or in combination with infrared technology. Lastly, far‐infrared technology was proposed as an appropriate technique for reducing the microbial load of black pepper seeds without affecting the qualitative characteristics (moisture content and volatile oils). Ha and Kang (2013) investigated the impacts of near‐infrared and ultraviolet irradiation on foodborne pathogens and the quality parameter of red pepper powder. Combination treatments over a 5‐min period resulted in a reduction of 2.78 and 3.34 log CFU *Escherichia coli* O157: H7 and *Salmonella typhimurium*, respectively. Moreover, no significant changes with respect to color parameters, and capsaicin and dihydrocapsaicin content were observed compared to the control sample (*p* > .05). Cheon et al. (2015) hired combined systems of ultraviolet (UV‐C) with a radiation intensity of 3.40 mW/cm^2^ accompanying thermal treatments of 25, 35, 45, 55, and 65°C for 5 and 10 min to cause inactivation of foodborne pathogens (*S. typhimurium* and *E. coli* O157: H7) in red pepper powder. The results showed that combined treatments (65°C and 3.40 mW/cm^2^) reduced the levels of *S. typhimurium* and *E. coli* O157: H7 to 3.06 and 2.88 log CFU/g, respectively. Though in ultraviolet alone (10 min at intensity of 20.4 kJ/m^2^), the decrease in these values was reported to be 0.29 and 0.22 log CFU/g, respectively. There was no significant difference between the color of the samples in the control sample and the samples treated with the combined system. Eventually, it was reported that the combined system is more effective than ultraviolet irradiation alone for decontaminating microorganisms without degrading the quality of the powder, and has been suggested as an alternative to conventional methods (superheated steam). Choi et al. (2018) examined the effect of the combined radiofrequency treatments (500, 1000, and 1500 W for 2 min) and cold plasma (700, 1000, and 1500 W for 2 min) on qualitative parameters and microbial characteristics of red pepper powder. In the combined system, the samples were first exposed to different radiofrequency powers and instantly placed in the refrigerator for 5 min. Finally, the samples were transferred into a cold plasma system. The results revealed that the combined radiofrequency system and cold plasma were more effective in reducing microbial load rather than individual treatments. In addition, the combined system led to minor changes in the color parameters of the samples. Eventually, it was reported that the best condition to reduce the microbial load (*S. aureus* counts and *E. coli* O157: H7 to 3.19 and 3.73 log CFU/g, respectively.) without significant changes in color parameter or antioxidant activity of red pepper powder was when using the power of 1500 W in the radiofrequency system and a power of 1000 W in the cold plasma system. Watson et al. (2020) examined the combination of ozone, ultraviolet, infrared, and fluidized bed systems for decontamination of chili flakes. Treatment with each method effectively reduced the microbial load to 6 logs (CFU/g) in ≤20 min for ozone, 7 logs (CFU/g) in ≤40 min for UV, and 7 logs (CFU/g) in ≤20 min for IR. The combination of infrared and ultraviolet treatments also improved performance compared to individual treatments. Finally, the combination of infrared and ultraviolet treatments followed by ozonation (UV and IR for 10 min and ozone 10 min) was reported to be an effective way to decrease the contamination in chili flakes; this is due to more effective reduction in microbial load for infrared and ultraviolet (0.80 log (CFU/g)) compared to ozone first (0.13 log (CFU/g)).

## 6 CONCLUSION

MAPs, like other agricultural products, are prone to contamination with germs, insects, etc. at any step in the production chain. Therefore, decontamination of MAPs with the aim of reducing adverse effects on raw material compounds, less waste, and more added value along with consumer health is necessitated. Fumigation with ethylene oxide and methyl bromide, heat treatment with steam, and gamma irradiation are common technologies that have been used on a commercial scale for the decontamination of MAPs. These technologies have disadvantages such as the formation of toxic by‐products and carcinogenicity, poor acceptance among consumers, and also the impact on the quality of the product. New technologies such as ozonation, cold plasma, ultraviolet, infrared, microwave, and radiofrequency have been used in order for decontamination of MAPs to overcome these limitations. These technologies have improved product quality parameters compared to conventional methods. However, these technologies are limited to a specific product (powder, seed, and leaf) and some of them have been used on a laboratory scale. Combined technologies have recently been studied for the decontamination of MAPs. It is to be hoped that with the development of such a system, a major step will be taken to ameliorate the quality control of MAPs and thus increase the added value of the product. Further research is required to develop the system, by taking into consideration environmental issues, the optimal relationship between product quality and microbial properties, energy consumption and continuous process. In addition, after the development of such a system, the obstacles that cause the transfer of this technology should be examined from the laboratory to the industrial scale, as well as the equipment required on an industrial scale and the initial economic evaluation of the process.

## CONFLICT OF INTEREST

The authors declare that they have no conflict of interest.

## ETHICAL APPROVAL

This study did not involve any human or animal testing.

## Data Availability

Data sharing is not applicable to this article, as no datasets were generated or analyzed during the current study.
